# Brexit, Covid‐19, and Possible Frameworks for Future UK/EU Financial Governance Cooperation

**DOI:** 10.1111/1468-2230.12650

**Published:** 2021-06-28

**Authors:** Elizabeth Howell

## Abstract

The EU project is at an inflection point. Intra‐EU alliances are altering following the UK's departure, the EU's financial markets remain segmented, and there is limited appetite for completing the Banking Union. The second stage of Brexit negotiations also collided with the Covid‐19 pandemic, which has strained economies around the world. These issues amount to a ‘polycrisis’ for the EU, raising existential questions about its future. This article focuses on one strand of the debates generated within this polycrisis: future UK/EU policy cooperation with respect to financial governance. The article discusses the importance of the financial services sector to the UK and the EU, and examines potential institutional options for future cooperation. In particular, it advocates harnessing dexterous aspects evident within precedents, including existing EU/third country association agreements, to develop a functional arrangement for future financial governance cooperation, which could also lead to closer UK/EU cooperation than currently appears likely.

## INTRODUCTION

The EU project is at an inflection point. Intra‐EU alliances are altering in light of the UK's departure,^1^ and the EU is grappling with a constellation of crises, including the continued fallout from the financial and eurozone crises, which has also spawned populist movements in some Member States. The EU's financial markets remain segmented and there is limited appetite for completing the Banking Union. The second stage of Brexit negotiations also collided with the unfolding Covid‐19 pandemic, a medical emergency that has placed economies around the world under tremendous strain.^2^ This crisis has consumed governments’ attention with respect to managing the spread of Covid‐19. It has also reinforced the primacy of the nation‐state in a time of crisis,^3^ and produced new divisions within the EU with respect to forming a collective strategy for tackling Covid‐19 and its economic fallout. The combination of issues amounts to a ‘polycrisis’ for the EU, aspects of which raise existential questions about the future of the EU project.^4^

This article focuses on one strand of the debates generated within this polycrisis: future UK/EU cooperation with respect to financial governance, or the mechanisms which support financial market regulation and supervision. In this regard, ‘cooperation’ can be defined in broad terms as any agreement or organisational arrangement, formal or informal, between countries to promote some sort of cooperation in the design, monitoring, enforcement, or ex‐post evaluation of regulation.^5^ The article discusses the importance of the financial services sector to the UK and the EU, reflects on the various financial governance crises facing the EU and explores how these may generate the space for cooperation.^6^

On the one hand, any ambitious and innovative developments may appear unlikely in the short‐term, especially given the various UK/EU political tensions that have arisen post‐Brexit regarding a series of issues (including the supply of the Covid‐19 vaccine to the Union; the flow of goods between Britain and Northern Ireland, and with respect to financial services).^7^ Moreover, the short‐term UK/EU vision regarding a future financial governance relationship refers expressly to establishing only ‘regulatory cooperation’ and that the parties will agree a non‐binding Memorandum of Understanding (which establishes a joint regulatory forum for the UK and EU to discuss financial services issues) to further such cooperation.^8^ Nevertheless the article suggests that there is greater scope for more dynamic solutions over the longer term than the current pessimistic consensus allows for. In particular, the article highlights the nature and the significance of the EU/UK interdependence in the field of financial services. Given this, the article shows that it remains in the mutual interest of the EU and the UK to develop a new financial governance relationship based on a high degree of cooperation between the two jurisdictions. Such a framework would enable the existing, deeply integrated cross‐border flows in finance to continue and would assist in sustaining employment and growth throughout Europe.^9^

Turning specifically to the EU, the article explores the various challenges facing the Union in the field of financial governance, and reflects upon how these may influence a new institutional framework. In particular, the EU is struggling to complete a number of ongoing projects, including its euro‐area Banking Union project. The future of its Capital Markets Union initiative also remains unclear. In addition, in the light of the UK's departure from the Union, intra‐EU alliances have shifted, and the Covid‐19 pandemic has introduced new disagreements between states as to how best to tackle its economic impact. Today's EU project is also notable for the level of variety inherent in it: some projects are single market endeavours whilst others are eurozone‐specific. Taking account of such variables, the article shows that the crises the EU faces, and the level of differentiation in the set‐up within and surrounding the EU could offer a crucible for experimentation.^10^

In this regard, the article considers three potential institutional options for future UK/EU financial governance coordination over the longer term based on the adoption of common standards. It explores the creation of a joint financial governance committee; the development of bilateral and mini‐lateral alliances (that could offer the UK some market access rights with respect to particular Member States); and a functional UK/EU association regarding the pursuit of a common policy on future UK/EU cooperation. In relation to a joint committee, the article suggests that there are risks as to whether this could achieve concrete results; its success would particularly depend on its status and objectives. With respect to bilateral and mini‐lateral initiatives, the article explores the UK/Member State relationships that are emerging and suggests that further productive transnational coalitions may develop over the longer term. Such arrangements could, for example, open up some aspects of Member State markets to the UK and could also indirectly impact upon a future UK/EU relationship and the parties negotiating positions in respect of such a relationship. Yet such strategies for enhancing cooperation are not a panacea. They require much negotiation and, given that different states will wish to advance different national interests, they can only advance a limited range of initiatives. Accordingly any such arrangements should be pursued in addition to formulating a new, functional UK/EU association. In relation to this, an examination of existing EU/third country association arrangements illustrates that these are not simply ‘off the shelf’ frameworks; they have the capacity to be surprisingly versatile.^11^ Drawing on precedents which illustrate that bespoke mechanisms can filter into such EU/third country frameworks, the article advocates a functional UK/EU arrangement to develop future financial governance cooperation. In the first instance, such a set‐up could be utilised to accelerate a recalibrated Capital Markets Union initiative. This EU project, which aims to create a true single market for capital, has become all the more vital in light of the Covid‐19 crisis. Specifically, all economies (including the UK outside the EU) are facing much higher levels of debt and many businesses will require substantial new forms of equity funding going forward.^12^ In this regard, a mutually beneficial UK/EU arrangement that facilitates firms’ access to finance could serve as a platform to further EU/UK financial governance cooperation. More broadly, the development of a new UK/EU relationship over the longer term will also enable the two jurisdictions to have a stronger communal voice on the international stage where many of today's new challenges lie.

The article's structure is as follows: after this introduction, the following section discusses the short‐term picture regarding UK/EU financial governance. It particularly focuses on the importance of the financial services sector to the UK and the EU in order to illustrate why it should be in the interests of both jurisdictions for the deeply interconnected, cross‐border finance flows to continue. The article then turns to examine the internal challenges the EU is facing, in order to illustrate that this creates the conditions for experimentation. Building on these sections, the article then examines three potential institutional structures for future UK/EU cooperation in the field of financial governance. The final section concludes.

## UK/EU FINANCIAL GOVERNANCE: A SHORT‐TERM SNAPSHOT

### The importance of financial services to the UK and the EU

Many reports document the importance of the financial services industry for the UK. London is a leading global financial centre, second only to New York in the 2020 Global Financial Centres Index.^13^ It is a key sector for the UK, contributing £132 billion to the UK's economy in 2018 (amounting to an estimated 6.9 per cent of total economic output), and with over one million people working in the sector.^14^ It is also a global asset, serving as a hub for providing financial services to counterparties from other countries. It provides wholesale financial services (including derivatives activity, bond and equity trading, foreign exchange transactions and commodities trading) to the EU sector and the wider economy.^15^ The high liquidity of London's capital markets, plus the network effects of having all services in the one place, also places London at an advantage over other EU financial centres. It is a financial ecosystem that allows for economies of scale and depth of capital market activity that cannot easily be replicated.^16^

In this regard, the EU's financial system, particularly the EU's wholesale market, has relied to a large extent on sophisticated services provided out of London; the EU has benefitted from the network effects of London as a global financial centre. The UK is often described as the EU's investment banker; it is estimated that almost 80 per cent of EU capital markets and investment banking revenue is transacted in the UK.^17^ Next, while the UK was an EU member, the EU was the second largest capital market in the world with 21 per cent of global activity.^18^ In contrast, without the UK, the EU will have a diminished global footprint: the UK's departure will result in a reduction of the EU's share of global capital markets activity to 14 per cent, (one third the size of the US and approximately the same size as China).^19^ In essence, post‐Brexit, EU capital markets will be smaller and less developed with the EU economy even more reliant on the banking sector.^20^ Finally, the overall financial services industry is also highly regulated and during the UK's membership, the EU passporting system enabled easy access for firms across the EU market. This has been a mechanism from which EU firms benefited as much if not more so than the UK firms.^21^

Taken together, the high level of EU/UK interdependence in this area is evident. Large volumes of cross‐border financial trade and operations occur between the two jurisdictions. Moreover, the UK financial services industry and the ecosystem advantages of the City of London (the City) offer an enormous amount to businesses and individuals based within the EU. In contrast, no single European centre dominates in every financial sub‐sector, making it very difficult to replicate the advantages which the City possesses.^22^ For example, even in the post‐Brexit environment, the City continues to control the bulk of the euro derivatives clearing market (where a third party acts as a middleman for derivatives contracts agreed between a buyer and a seller). The UK's legal system, the City's expertise, and the scale of its financial markets all make it a very attractive location for market participants.^23^ Moreover while the Union is keen to bring this business to the continent (where there could then be the splintering of business between different European centres), it is struggling to persuade firms to move voluntarily.^24^

Accordingly, with such aspects in mind, and as the article will explore, it should be in the interests of the EU, the UK, and the financial services industry, to retain the existing, highly intertwined, cross‐border flows between the two jurisdictions. Avoiding financial fragmentation should be important for both the EU and the UK, and creating a new framework based on a high level of UK/EU regulatory (and ideally supervisory) cooperation will be necessary to provide a solid foundation in order to continue cross‐border activity.^25^

### Brexit: Short‐term financial sector measures

As has been pointed out ‘[f]or much of the City, Brexit happened sometime last year’.^26^ Specifically, many of the global entities that provide investment banking services are from the US (the so‐called ‘big five’). These banks have generally used the international financial centre of London as its main ‘hub’, with ‘spokes’ based in other mid‐sized and large European centres.^27^ Prior to Brexit, banks and other financial firms were proactive and took steps, including through setting up offices in EU cities (although, since the advent of the Covid‐19 pandemic, several financial institutions delayed or revised their plans to move staff to European cities).^28^ Some European banks also opted to merge their UK entities with their EU entity (which will have passporting rights) in order to grant them flexibility. Indeed, where financial institutions have reacted to the loss of passporting rights by, for instance, setting up an EU subsidiary or through merging with their EU entity, then they will remain subject to EU law. For such businesses, there therefore will be far less anxiety about whether or not the UK and EU reach a future financial services agreement (although such firms will remain concerned that the UK does not diverge from EU law in order to avoid subjecting their business to dual regulation).

With respect to relocation destinations, recent analysis has suggested that Dublin is the top choice overall (followed by Luxemburg and Frankfurt). However, as predicted by the discussion above, the landscape is more multi‐polar and fragmented than before, with firms moving staff and businesses to more than one European centre.^29^ While the precise level of job moves from London to another European city, as well as causes and effects, is open to debate;^30^ a 2021 report identified almost 7,600 expected staff moves (up from 7,500 in October 2020).^31^ Taken together, relocating staff constitutes contingency planning by institutions due to the loss of passporting rights and indicates that firms will adjust as they see fit in order to operate effectively in the post‐Brexit environment.

The UK's financial regulators have also taken many Brexit‐related precautions.^32^ To date, the UK approach can be broadly characterised as one geared towards providing certainty and facilitating access for EU firms.^33^ For example, a range of transitional licenses and exemptions were compiled to ensure that a wide range of financial services could continue to be offered by inbound EU providers in the event there had been a no‐deal situation, and the UK's temporary permissions regime still came into effect at the end of the 2020 transition period.^34^ The UK also granted a package of equivalence decisions to European Economic Area (EEA) states, including the EU Member States, to provide clarity and stability to the industry.^35^

Turning to the EU, in contrast to the UK, it largely did not provide for reciprocal short‐term access regimes in the event there was to be a no‐deal scenario. It repeatedly emphasised that individual businesses and Member States were responsible for taking the necessary precautions for any future no‐deal outcome.^36^ In addition, with a view to attracting business in light of Brexit, guidance was provided for firms relocating to the EU.^37^ The EU's more inflexible stance to UK market access rights can be understood as a facet of the EU's broader approach during the Brexit negotiations; the quid pro quo for full internal market access is abiding by the four freedoms.^38^ In particular, the EU did not wish to set precedents that other closely connected third countries could then demand.^39^ Essentially, in its dealings with third‐countries, the EU's policy has been focused on protecting its own interests and the preservation of the integrity of the single market. In this regard, the UK (or any other third‐country) that does not meet the obligations of a member, cannot enjoy the same benefits as a non‐member. This approach was reiterated in 2020, when the European Commission (the Commission) outlined preparatory discussions on the future UK/EU relationship. This underlined that EU equivalence assessments are unilateral decisions, and that the EU should make such assessments in protection of its own interests.^40^ The impact of the EU's firm stance also played out clearly in the early post‐Brexit days when, due to the absence of a UK equivalence decision being granted by the EU, Amsterdam overtook London as Europe's largest share trading centre.^41^

Taken together, this section illustrates that, on the one hand, the City will always adapt; it will find a way around rules and frictions. It also demonstrates that the EU will, by in large, retain its tough stance in its approach to third‐countries. Accordingly, looking to how the next stage is likely to unfold, and as the article will discuss, future UK/EU cooperation arrangements which reflect the new reality will be necessary.^42^

## THE CONSTELLATION OF CRISES FACING THE EU

### The EU's polycrisis and the future of EU integration

Aside from the external‐facing aspects of the UK's departure, the EU is confronted with its own range of internal crises with respect to financial and economic governance. This section considers these to demonstrate (as the following section then explores) that such challenges can generate the space for experimentation, even when politics can seem to render this hopeless. Further, with respect to the possible institutional options for future cooperation, there is much scope to work with the level of differentiation that now characterises and surrounds the EU project.

In relation to the EU's current problems, the Union is still struggling to complete the post‐2008 financial crisis reforms process. In particular, the effects of the eurozone crisis are not fully resolved. The crisis contributed to the emergence of nationalist and populist political groups, and political capital and trust in the eurozone states is at a much lower ebb than it was before the global and eurozone crises hit. Most recently this has been witnessed in the context of the pandemic where the EU has faced considerable criticism, including regarding its slow rollout of its coronavirus vaccine programme.^43^

More broadly, intra‐EU alliances have altered in light of the UK's departure,^44^ and, the Covid‐19 emergency has reiterated the deep divisions that exist between particular Member States with respect to agreeing common strategies for tackling its economic impact. For instance, ideological faultlines were on display regarding proposals from some Member States to issue ‘coronabonds’ (essentially collective debt) to finance the response to the crisis.^45^ Although a compromise EU deal fund was ultimately secured, the final agreement was considerably pared back from the initial visionary proposals due to vociferous objections from a number of northern Member States.^46^

Brexit percolates into many of these issues, raising deep questions about the future of the EU's project.^47^ As with the election of President Trump in 2016, Brexit bucked the trend towards greater globalisation and increased political integration.^48^ The UK's departure placed the EU project at an inflection point, a situation now exacerbated by Covid‐19.^49^ Indeed, as well as accentuating the question regarding the future relationship between the euro and the non‐euro states (an aspect explored later in this section), Brexit could also generate risks of further disintegration, it could equally be a means of advancing future integration.

Undoubtedly however, initial fears that there could be a Brexit contagion effect^50^ have not materialised; and this was likely assisted by the UK's difficult Brexit negotiations, as well as the significant economic costs anticipated to follow from the UK's departure (already in evidence in early 2021).^51^ With respect to Brexit generating calls for greater integration, there have also been suggestions that the EU requires more integration ‘of the right kind’ to transform the EU into a genuinely confederal model.^52^ Yet taking a step back, it is likely too soon to know whether or not Brexit is merely a blip on the way to greater EU integration. Further, this need not be a binary ‘disintegration or further integration’ choice; a range of more intermediate scenarios are possible including the EU project stagnating, or, perhaps most likely, a period of ‘muddling through’.^53^ Connected to this, Covid‐19 creates a new existential moment for the EU. On the one hand, nation‐state responses were inevitable; as Eidenmüller discusses, the EU's formal role on health issues is nominal, plus states can learn from the experience of others, which can lead to ‘coordination by imitation’.^54^ Yet this is a delicate issue, nation‐focused approaches can risk feeding into pre‐existing nationalist‐populist movements.^55^ Further, as identified above, the pandemic created new rifts with respect to approving an EU‐wide economic response.^56^ There have also been various criticisms from Member States (including Ireland) regarding the EU's sluggish rollout of the vaccination scheme.^57^ Yet recent events also point to the EU's ability to broker deals and find solutions to formal problems.^58^ Indeed while the pandemic has posed new tests for the Union, the EU's deep survival instinct has also kicked in to ameliorate any risk of disintegration.^59^

### A changing Europe: Deepening economic and monetary union

Despite the various tensions, the Commission's longer‐term vision involves moving towards strengthening the Union's Economic and Monetary Union (EMU). This includes completing the Banking Union, and expediting the Capital Markets Union.^60^ Yet, connected to the analysis above, obstacles were not simply of the UK's making; there are divisions between and within Member States over the structure and the desirability of greater integration.^61^

### Banking union: Stuck in the mud?

The eurozone's incomplete Banking Union project is a good illustration of the ongoing disagreements. Italy's initial emergence as the European centre of the Covid‐19 virus (in the first wave) revived concerns regarding the strength of Italy's banks, illustrating that the ‘deadly embrace’ between banks and sovereign states has not yet been resolved.^62^ In the eurozone, this vicious circle creates a risk of contagion; countries sharing a currency through monetary union are interdependent, so problems that originate nationally can risk affecting other countries and may affect the stability of the whole of the eurozone.^63^ The Banking Union was geared towards tackling this by removing these close connections through centralising supervision and resolution.^64^ Its final pillar is intended to be a European Deposit Scheme, however this remains the project's missing link. A sharp dividing line remains between countries with more stable banking regimes (such as Germany's) who could be net contributors to the scheme,^65^ and where their banking systems (and their depositors) could be guaranteeing depositors of banks in less stable regimes (such as Italy's).^66^

The project is also a valuable illustration of intra‐EU coalitions shifting in the post‐Brexit landscape. When the Banking Union's architecture was being negotiated, the UK as a non‐participating non‐euro state, acted as an important voice for the other non‐euro states^67^ to ensure their interests were protected.^68^ Accordingly, the loss of the UK's key voice now alters the political balance of power within the EU.^69^ At first glance, this could result in greater integration in the eurozone (centripetal effects) whilst the distinction is increased between the core and the euro‐outs (centrifugal effects).^70^ Yet, the post‐Brexit geometry of power is also becoming more complex. To provide conceptual clarification to the messier reality,^71^ two groupings can be identified. There are the states focused on greater risk‐sharing and greater fiscal transfers, such as France and Italy. In contrast, the other group, which traditionally included Germany and the Netherlands are focused on reducing risk, stricter implementation of rules and economic self‐responsibility.^72^ In light of Brexit, some of the states within the second grouping have developed a new alliance, the so‐called ‘New Hanseatic League’. This is composed of eight northern economically liberal and fiscally conservative Member States.^73^ Its post‐Brexit emergence can be regarded as an attempt to maintain a competitive agenda in the Union, and to counter France and Germany's enhanced role. For instance, the New Hanseatic League is broadly opposed to far‐reaching transfers of competence to the supranational level, but advocates the deepening of the single market, including (as considered below), the further development of the Capital Markets Union (CMU) initiative.^74^

### What should happen to the CMU?

Turning to the CMU, it remains an open question to what extent the EU can continue to push forward with this post‐Brexit. When the Commission compiled its CMU plans (that broadly aims at strengthening access to market‐based finance, whilst reducing reliance on bank financing), the UK was intended to be a part of it, with its integral importance in and stimulus to the EU's capital markets.^75^ As articulated earlier in the article, the City's success is linked to a successful Europe.^76^ The UK hosts the EU's largest capital markets and in some market segments the UK accounts for up to three quarters of EU activity.^77^ In contrast, the EU markets are decentralised and no single location offers a close substitute for London. Further, although Brexit has clear short‐term effects for the UK's financial industry, London is unlikely to easily lose its prominent position as a key financial centre.^78^ More generally, the CMU has not proven to be a major force in the reconfiguration of EU and global markets following the 2008 financial crisis. Rather, when access to traditional finance became difficult, other areas such as ‘fintech’ stepped in to fill companies’ financing gaps. In this regard, it could even be the case that weaknesses of the CMU further facilitated this process.

Despite its deficiencies, the Commission wishes to accelerate this project^79^ and given that there is little energy for completing the Banking Union, completing the easier‐to‐achieve CMU could best strengthen the EMU. Further, although the project has lost some of its original vision, it can still move forward, albeit in an adapted form. Financial market activities benefit from concentration in a single hub and so it could be to the EU's advantage to leave this system broadly intact.^80^ Given this, the CMU could be a valuable initiative to further UK/EU financial governance cooperation over the medium‐to‐longer term. Indeed, as it is probable that both the UK and the EU will be recovering from the economic hit brought about the Covid‐19 pandemic for some time, the CMU can be viewed as all the more crucial. Accordingly, a mutually beneficial UK/EU arrangement could enable firms’ easy access to market‐based finance (thereby tempering debt levels) via their coordinated efforts.

Overall, the section has illustrated the polycrisis facing the EU as well as the heterogeneity inherent in the EU project today. Such a setup can pave the way for blue sky thinking, even when the current political landscape may suggest otherwise. This then connects to the related aspect, discussed in the next section, as to which possible frameworks could be best utilised to develop future UK/EU financial governance cooperation.

## OPTIONS FOR UK/EU FINANCIAL GOVERNANCE COOPERATION

### Scope for experimentation

The questions generated by the confluence of CMU and Brexit can be boiled down to how open the EU capital markets should be to the world post‐Brexit, or whether it should preserve the status quo to gain a competitive advantage over the UK.^81^ As discussed in the article's earlier sections, the short‐term indications point to the EU signalling the integrity of the single market and highlighting that third country rights of access are inferior to being part of the EU club.^82^ At first blush, there is also no evidence of new thinking emerging from the EU on third country access rights.^83^ Indeed, over the years, a combination of path dependence plus lock‐in effects have very much conditioned the thinking of EU leaders. Specifically, the EU has a preference for using pre‐existing models; moreover, once a ‘solution’ is reached, lock‐in arguments mean that other alternatives have been rendered implausible.^84^ Further, although the EU should have much interest in a close future relationship given the deep and long‐standing interconnections that exist between the two jurisdictions in the financial governance sector, the EU does not wish to set any precedents with a bespoke EU/UK arrangement that other Member States or closely connected third countries (such as Switzerland) could then request.^85^ Moreover, as explored in the article's earlier sections, the adoption of a tough stance could also enable various EU hubs to benefit from financial institutions relocating from London.

Be that as it may, the reality is more nuanced. Over the decades, there are precedents illustrating that the EU has reached accords with various closely connected jurisdictions to grant internal market access rights (to varying degrees). On the one hand, these may apply common provisions in particular policy sectors, while also permitting some barriers to cross‐border access. Indeed, even where the texts in question use the same terminology as EU law, in application the agreements are heterogenous and context‐specific.^86^ For instance, in the EEA Agreement, which broadly applies EU law to the EEA states, there are important carve‐outs for fisheries and agriculture.^87^ Likewise, the association agreements in place with Ukraine and Georgia provide a high degree of access rights for goods, services and capital, whilst carving out labour.^88^ A further salient illustration is with respect to Swiss‐EU relations, which have historically been governed by a complex matrix of bilateral sectoral agreements where EU rules apply to particular areas, with various exceptions that traditionally included financial services.^89^

Indeed despite suggestions that the EU struggles to dynamically adapt, the EU has sometimes embraced ‘creative solutions to formal problems’,^90^ prioritising political and economic considerations over legal impediments. Euro‐crisis innovations such as the Banking Union, involved considerable exercises in ‘legal gymnastics’,^91^ as did the creation of the EU financial sector agencies.^92^ Some bespoke elements also appeared in the first wave of Brexit negotiations with respect to particular policy issues (including in relation to Northern Ireland, and the demands of the fishing industry), where it was felt that the EU's interests were best served by ‘doing things a bit differently’ than before.^93^

All such precedents demonstrate that there can be capacity for new thinking and legal creativity, particularly in response to crisis situations. Connected to this, and as evidenced in the above section, the EU endeavour today is defined by a complex matrix of projects that apply to different states. Some (such as the Banking Union) apply only to the eurozone (albeit with opt‐ins); and others (such as CMU) are single market endeavours.^94^ While there are no silver bullets, there is scope for experimenting with the variable geometry which now characterises the EU project.^95^

### Options: Experimenting with differentiation

This section explores possible frameworks for future UK/EU regulatory coordination through the adoption of common standards, which (if the political will exists) could also extend to embrace supervisory cooperation arrangements.^96^ Indeed, the UK has recently acknowledged the importance of international coordination for financial services, stating that an ‘optimally designed regulatory framework should … facilitate cooperation and the development of common standards across international regulatory bodies and jurisdictions’.^97^ In terms of how such an arrangement could be implemented, there are a number of possibilities. For instance, common standards could be implemented via an agreed voluntary framework of principles; there could be a midway model that combines so‐called ‘hard principles’ with sanctions imposed in the event of non‐compliance; or there could be the introduction of binding legislation. Again, the precise structure chosen will be dependent on the political consensus, including both sides’ level of ambition with respect to their future cooperative relationship.

While this section focuses on the potential options for future UK/EU cooperation arrangements, it should be borne in mind that (although beyond the scope of the article) there are also market‐based solutions that could be utilised, and which could facilitate various market access rights between jurisdictions without the need for regulatory convergence. For instance, the Stock Connect scheme is a recent programme that links the Hong Kong and Shanghai stock exchanges, as well as the Hong Kong and Shenzhen Stock Exchanges. Such schemes can enable mutual market access, deepen cooperation and communication between the stock exchanges and enhance the competitiveness of the respective markets.^98^

With respect to potential institutional frameworks, three possible options for future coordination via the endorsement of common standards are examined: a joint committee for financial governance cooperation; the establishment of bilateral and mini‐lateral alliances that would offer the UK some access rights to particular countries; and a functional arrangement for future UK/EU cooperation. This section suggests that committees are not a panacea; and that while bilateral and mini‐lateral alliances are imperfect and limited solutions, they could offer some market access and potentially strengthen the UK's negotiating position with respect to a future UK/EU partnership. In relation to a functional UK/EU financial governance association, on the one hand, the signs do not augur well in terms of its short‐term feasibility. Specifically the Trade and Cooperation Agreement which was concluded between the UK and EU in December 2020 (the TCA)^99^ does little to enable access to the single market in relation to UK financial services (these were largely excluded from its ambit).^100^ On the other hand, as identified earlier in the article, under the non‐binding Joint Declaration on Financial Services Regulatory Cooperation (Joint Declaration), the UK and EU commit to establishing regulatory cooperation in financial services. This is to be facilitated by a Memorandum of Understanding (and which creates a joint forum to promote dialogue on financial services issues).^101^ While the Joint Declaration is non‐binding in form and is lacking in granular detail, this can also be regarded as a starting point from which a future relationship starts to build. With this in mind, it is suggested that it would be in both sides’ interests to ultimately secure a new and innovative financial governance cooperation arrangement. This is particularly the case given the nature of the UK and EU's interconnectedness in this vital policy area and bearing in mind their close geographical proximity.^102^ Accordingly this section suggests that a mutually beneficial functional compact should be achievable over the longer term.

In this regard, inspiration can be drawn from existing EU/third country association agreements, as considered above. On the one hand, analogies cannot be taken too far; association agreements aim at convergence whereas Brexit concerns divergence and less political cooperation.^103^ Yet, association agreements remain relatively flexible instruments that could be adapted to the particular UK/EU circumstances, and could contain special provision for financial services.^104^ Such a framework could be used to develop a simplified and enhanced UK/EU market, including, in the first instance, expediting a rewired CMU project in a non‐zero sum form.^105^ As this section discusses, while the system would need to recognise the parties’ individual regulatory and decision‐making autonomy (part of the UK/EU 2019 New Political Declaration but also a UK red‐line),^106^ the UK and the EU could commit to using best endeavours to maintain mutual access in the field of financial governance as far as possible. There would also be a commitment to continued close regulatory (and potentially supervisory) coordination. This could also include future limited UK participation in the EU financial sector agencies and UK/EU coordination in the international financial governance sphere.^107^

### A joint committee

The first option would be to set up a dedicated joint high‐level committee to mitigate the shock of Brexit to financial services in the EU. For instance, the European Shadow Financial Regulatory Committee (a body comprised of experts from private organisations and academic institutions that analyses policy issues as regards the financial services industry) has suggested that such a committee could develop proposals for simplified and better regulation that would strengthen market discipline.^108^ Such a committee would include the major financial authorities in the UK and EU (the Prudential Regulation Authority (PRA) and the Financial Conduct Authority (FCA) on the UK side; and the ECB (which oversees the Banking Union's Single Supervisory Mechanism), and the European Supervisory Authorities (ESAs) on the EU's side).

Reflecting on its potential powers, while there would be recognition of each side's regulatory autonomy (and an acknowledgment that equivalence assessments are unilateral decisions (an EU red‐line)), there would be a commitment to use best endeavours to secure mutual access. The committee would participate in drafting new laws, including making proposals with respect to developing the regulatory regime, and, (in principle) would also commit to information‐sharing, and supervisory and enforcement cooperation.^109^ In this regard, there could be a commitment to coordinate in relation to the joint oversight and enforcement of the markets.^110^ Further, in order to protect the EU's interests, especially concerns about future UK regulatory divergence, the committee's remit could include considering cases of potential divergence and whether such divergences should be referred to dispute resolution procedures. Given this, adopting a framework which embraces hard principles with penalties for non‐compliance could be valuable: this could both acknowledge the requirement for regulatory autonomy, whilst providing remedies with respect to any breaches.

In relation to the level of its formation, such a committee could be created bilaterally. For instance, this dedicated committee could be modelled on the existing joint UK/EU committee as laid down in the UK/EU 2019 New Withdrawal Agreement (New Withdrawal Agreement), which envisages the possibility of delegating to specialised committees.^111^ Similar joint committees can be witnessed in other EU/international agreements such as the EU‐EEA Joint Committee in the EEA Agreement,^112^ and the EU‐Canada Comprehensive and Economic Trade Agreement (CETA), which expressly establishes a Financial Services Committee as a specialised committee.^113^ This model is also included in other non‐EU agreements, such as the free trade agreement between the European Free Trade Association (EFTA) and the Ukraine.^114^

Alternatively such a committee could be formed at the international level of financial governance via the international standard setting bodies (ISSBs), such as the Financial Stability Board (FSB) and the International Organisation of Securities Commissions (IOSCO). The ISSBs implement high‐level political directions stemming from the G20 and are typically made up of national regulatory authorities or central banks, as well as containing EU representation.^115^ Whilst one must remain mindful of the different context and focus of the ISSBs, at first glance, a committee operating within an international avenue could seem preferable to a UK/EU committee due to its neutrality. At the same time, while the UK will still be a member of the major ISSBs post‐Brexit, it will no longer be a member of the ESAs and it will lose the additional platform for diffusing preferences that the ESAs provide on the ISSBs.^116^ Moreover, it could be tricky to orchestrate a committee at the international level that was merely comprised of EU and UK competent authorities. Accordingly, a bilateral UK/EU committee may be more appropriate, and the UK may wish to follow the model which is provided for in CETA or the UK TCA rather than to, for instance, embrace the joint committee model articulated in the 2019 New Withdrawal Agreement. For instance, CETA and the TCA contain dispute resolution provisions that focus on an arbitration model plus channels for political agreement, rather than there being a devoted role for the Court of Justice of the European Union (CJEU).^117^

Further, aside from the questions concerning its model and level of operation, how beneficial any such committee would be is debatable; much would ultimately depend on its precise status and powers. As identified above, ideally such a committee would be created to develop regulatory proposals and to commit to oversight and enforcement cooperation via a framework that provided for remedies in the case of non‐compliance. Yet, its precise parameters would ultimately be dependent on the level of political consensus. Moreover, what (yet) another committee may be able to achieve in what is already a committee‐heavy financial governance system could be limited in practice. Indeed, committees can generate considerable work for the participants yet may not be able to actually deliver much in the way of results.^118^

### Bilateral arrangements

With respect to bilateral arrangements, much of EU law contains non‐discrimination provisions meaning non‐EU firms cannot be treated more favourably than firms from Member States. Subject to this, however, bilateral agreements are a possibility in the spaces where the EU has not harmonised third country treatment. For example, under the Markets in Financial Instruments (MiFID II) regime, Member States can allow third country firms to provide investment services in their territories in the absence of the Commission making an equivalence decision.^119^ At the time of writing, as there is no Commission equivalence decision in place for the UK, it is open to the UK to reach agreement with particular countries so that UK firms can obtain access to wholesale customers in those states. Equally under EU banking legislation, Member States (and the ECB within the Banking Union euro area) have the discretion to allow third country banks to establish branches in its territory, provided the rules are not more favourable than those applied to other Member State branches.^120^ Moreover, with respect to collective investment funds, under the Alternative Investment Fund Managers Directive (AIFMD), there is the ability for third country alternative investment funds (AIFs) to be marketed to professional investors in a Member State, subject to certain conditions.^121^ While this particular system is to be phased out once a ‘third country passport’ regime becomes available, at the time of writing the third country passport is still to be activated. In this regard, there is also a notable bilateral precedent within the EU from 2014 between Germany and Switzerland that facilitates the distribution of particular German funds in Switzerland, and of Swiss securities funds in Germany (with the Swiss funds being regarded as third country AIFs under EU law and with restrictions in place regarding the marketing of fund units).^122^

Comparatively, and at a regulator to regulator level, bilateral Memoranda of Understanding (MoUs) are regarded as particularly plausible mechanisms for successfully promoting cooperation, and are now a common tool used by regulators, especially with respect to information exchange.^123^ Indeed many of these MoUs supplement IOSCO's Multilateral MoU, which is regarded as the benchmark for international cooperation and sets a floor for minimum obligations.^124^ In the US for instance, the SEC makes extensive use of cooperative MoUs with foreign regulators,^125^ using them to effectively increase information gathering through providing an information‐exchange framework.^126^ These include MoUs with respect to supervisory cooperation and enforcement assistance, and in relation to the provision of technical assistance. For example, in 2019, the UK and the US signed an updated MoU with the SEC, which was originally signed in 2006.^127^ The MoU is a comprehensive supervisory arrangement and it ensures the continued ability for the regulators’ to cooperate and consult with each other regarding the effective and efficient oversight of regulated entities across national borders.^128^ The MoU has also been expanded to reflect post‐financial crisis reforms related to derivatives, and to reflect the FCA assuming responsibility from ESMA for particular oversight responsibilities in light of Brexit.^129^ More generally, the use of MoUs today is part of a global trend. For instance, since 2008, the Australian regulator (ASIC) has been estimated to have entered into MoUs and other cooperative arrangements with more than 50 regulators, including with the FCA, various US regulators, and the Ontario Securities Commission in Canada.^130^

The flexibility inherent in MoUs’ soft law nature coupled with effective communication between authorities generally enhances the cooperative prospects.^131^ Brummer also notes that, given their non‐binding, soft law status, their effectiveness relies on the fact that regulators (most likely) want to stay in good standing with their peers and thus will comply with the terms of the arrangement.^132^ Further, even when parties enter into a MoU partly because it is unenforceable, the agreement's existence still tends to facilitate more cooperation than would otherwise occur, leading to surprisingly effective relationships between diverse authorities.^133^ Such elements are particularly significant in the context of Brexit given the immense political barriers which currently exist to agreeing a future binding framework.^134^ Accordingly, as is discussed later in the article, a non‐binding new financial services arrangement could still lead to a functioning and productive future UK/EU partnership.

Nonetheless, bilateral relationships also remain imperfect options; they require considerable negotiation, increase costs, and only a few elements can be prioritised.^135^ Yet the possibility of the UK entering into bilateral arrangements with individual states and regulators in addition to, or as part of a future UK/EU partnership, should not be dismissed. As explored above, where the EU has not harmonised third country treatment (such as with respect to the MiFID II regime, third country bank branching, and third country AIFs) this could open up aspects of the EU market to the UK.^136^ As well as tackling non‐harmonised areas, bilateral compacts could also strengthen the UK's position with respect to a future UK/EU alliance. For instance, this might especially be the case where particular proposals garner support from some but not all Member States.^137^

Empirically, some skeletal UK/Member State relationships are already emerging in the UK/EU financial governance zone. In February 2019, the FCA agreed MoUs with the EU (and EEA) national regulators covering supervisory cooperation, enforcement and information sharing, plus a MoU with ESMA concerning credit rating agencies and trade repositories (where ESMA has direct oversight responsibilities). Such MoUs provide for continued close cooperation, and support the cross‐border oversight of firms. While they were initially intended to apply in the event of a no‐deal Brexit scenario, the FCA and ESMA subsequently confirmed they would come into effect at the end of the Brexit transition period.^138^

More specifically, in response to the UK's temporary permissions regime (as explored earlier in the article), a number of Member States (as well as the EEA) implemented their own version of temporary measures that were to apply in a no‐deal scenario. While a number of these measures were suspended after the EU and the UK approved the 2019 New Withdrawal Agreement, some jurisdictions kept measures in place. While the picture is a varied one, and there are no silver bullets, a number of Member States have facilitated some access for UK firms. For example, as regards Finland, UK firms were encouraged to rely on the Finnish third country licensing regime. Where firms applied before the end of the Brexit transition period, UK firms received temporary permission to continue to offer and provide investment services in Finland until their application has been processed.^139^ Further, the Dutch regulator also extended an exemption (on a temporary basis, and which expired on 1 January 2021) to the UK that already applies to Switzerland, the USA, and Australia. This allowed UK‐based investment firms to provide investment services to professional clients,^140^ without requiring a local Dutch license. More broadly, however, the FCA and the Dutch Financial Markets regulator also agreed to formalise a broader partnership that would apply whether or not a UK/EU deal was reached. While the precise details have not been published, the partnership is due to Brexit, and reflects the fact that both economies are closely integrated with major flows of services and goods between the two.^141^ Indeed, several firms operating in both countries have applied for licences to continue to operate in the respective countries. The information that is available points to a focus on close cooperation and information sharing, and an alliance that builds on the relationship the two regulators already have with respect to fintech, data‐led supervision, and market abuse.^142^

There is also scope for further bilateral arrangements to arise. For instance, suggestions have been advanced that, if the UK were receptive, a future Sweden‐UK bilateral relationship could be contemplated. Again, this could be helpful for the UK as it navigates its new non‐EU status given that Sweden has valuable prior experience regarding close relationships with other non‐EU states.^143^ For example, Sweden has built long‐standing and close associations with non‐EU countries such as Norway (and it has operated in forums such as the Nordic Council which has both EU and non‐EU members). Given this, Sweden could (if the UK were receptive) use this knowledge to assist the UK in developing new collaborations in the post‐Brexit environment. More generally, even where there are notable restrictions in a Member State with respect to financial services’ market access, domestic regulatory changes within that jurisdiction could make the difference.^144^

All such examples highlight that there is capacity for bilateral UK/Member State alliances to emerge going forward in relation to future regulatory and supervisory cooperation. As identified above, the question of precisely who makes the decision as to whether third country firms can access EU markets is not yet standardised. Given this, in the absence of a Commission‐led equivalence decision that confers third country access, such decisions will rest at the national level. Undoubtedly, a more uniform EU approach to equivalence access has been developing in more recent years in some aspects of financial governance legislation explored above (including the proposed third country passport in the AIFMD regime and within the MiFID II regime). Nonetheless, at the time of writing, the AIFMD third country passport has not been switched on yet, and no UK MiFID II equivalence decisions have so far been made by the Commission (likely due to concerns about preferential treatment). Accordingly, as discussed above, Member States may still allow firms to provide cross‐border investment services in the absence of an equivalence decision. Equally, while it should be acknowledged that the EU has been critical of the particular set of bilateral agreements that have historically governed EU/Swiss relations (where Switzerland has selectively applied aspects of the EU acquis),^145^ the EU is not, as a rule, averse to the use of bilateral cooperation agreements. Indeed, the Commission recently noted that the international G20 framework is underpinned by a network of bilateral arrangements at the regulatory and supervisory level, and that these can contribute to the building of mutual understanding and trust between jurisdictions.^146^

The value of future UK/Member State bilateral relationships in this area is also particularly apparent when one keeps in mind the deep UK/EU interdependence in the financial governance arena (articulated earlier in the article), and London's position as the hub of EU capital markets. In this regard, it is also notable that there are wide variations among the Member States in terms of their market size and with respect to their capital markets development. Specifically, Europe has traditionally been especially reliant on bank finance, and there are considerable differences in capital markets development across the Union. For example, the Commission reported that domestic stock market capitalisation exceeded 121 per cent of GDP in the UK in 2013, compared to less than 10 per cent in Lithuania, Latvia, and Cyprus.^147^ Accordingly, going forward, the establishment of new bilateral relationships with the UK could be particularly beneficial for smaller markets (such as Finland and the Baltics)^148^ and for Member States with underdeveloped capital markets (such as the Baltics and Cyprus) that are especially dependent on bank finance. Such states may have a particular interest in firms and individuals having continued access to the City's ecosystem.

### Mini‐lateral alliances

A connected aspect to explore concerns mini‐lateral alliances, which are also on the rise today. Mini‐lateral alliances can arise in a wide range of policy areas and may come in many different forms, but broadly, these types of grouping can be regarded as joint ventures of interested parties with shared interests. For example, Brummer chooses to include the ‘(shaky) EU’ within the umbrella of mini‐lateralism, as well as EU mini‐lateral initiatives such as the Banking Union and the proposed Fiscal Union.^149^

Mini‐lateral clubs may be modest in size, but such coalitions can work together in order to supplement or complement the activities of international organisations.^150^ They can aspire to coordinate different aspects of the international economy and to export policy preferences of member governments.^151^ These groupings can be regarded as a relatively new and flexible form of cooperation, which can reach agreement more quickly, and (potentially) attain more ambitious outcomes than multilateral forums.^152^ Moreover, such clubs can cooperate using a variety of formats, ranging from the introduction of binding legislation (such as with respect to the Banking Union project) to the implementation of soft law, voluntary frameworks.

Such developments can also be particularly understood when reflecting on the fact that international, multilateral institutions such as the World Trade Organisation and the United Nations are all facing legitimacy challenges.^153^ Specifically, multilateral institutions are comprised of diverse groupings of states with particular world views and objectives. Given this, multilateral organisations can struggle with cumbersome frameworks as well as slow decision‐making structures that can result in watered‐down outcomes.^154^ Such institutions may also fail to dynamically respond to emerging challenges, illustrated most recently with respect to the obstacles in developing coordinated international or regional responses to Covid‐19. Accordingly, all such issues can result in alternative and innovative types of cooperation emerging. This can embrace the bilateral models discussed above, as well as mini‐lateral alliances.

As observed above, mini‐lateralism can arise in various guises but such arrangements and deals are generally ad hoc, modest in size and are based on shared interests and values.^155^ These arrangements are (again) not a panacea; such frameworks can be less inclusive, can lack legitimacy and can lead to a duplication of effort.^156^ Yet, applied to the post‐Brexit landscape, and drawing inspiration from the cognate political economy scholarship, more flexible transnational (or supranational) coalitions involving EU partners could gain greater traction over the longer term.

Such alliances (which also take account of the literature on ‘new interdependence’) connect to how global firms organise at the EU level to shape EU policy. Disaffected actors can forge alliances across countries and can have new avenues of agency that may disrupt the status quo.^157^ As EU financial integration is a reflection of the interests of large financial firms whose business is now pan‐EU, such firms will be impacted by reduced market access and it could be the case that alliances mobilise in favour of the UK retaining access to the EU single market.^158^ Indeed these relationships could also offer some scope for indirectly influencing the future development of UK/EU financial governance arrangements.

Admittedly, during the short term, the development of mini‐lateral coalitions of common interest may look unlikely. In particular, neo‐mercantilist battles have been playing out among Member States in relation to promoting their financial hubs and luring business away from the UK.^159^ Additionally, the City also failed to influence the Brexit policy in the shorter term due to a number of factors, including the downgrading of its interests due to the demands of the Conservative Party in crafting a new electoral winning strategy, as well as heterogenous preferences within the City itself about the implications of Brexit.^160^

Yet although mini‐lateral groupings have not to‐date materialised to any notable extent, taking the longer view, it could be feasible for such ad hoc arrangements to start to develop. For instance, large financial institutions which have considerable UK/EU cross‐border business operations could form a coalition advocating a special framework for future financial governance cooperation. Indeed, this may now be even more likely in light of the Covid‐19 effects. Moreover, in addition to this, such initiatives could also build and expand upon existing and future UK/Member State bilateral alliances. For instance, as explored above, as well as the relationships that have already materialised, new bilateral arrangements could be particularly valuable for jurisdictions that are currently over‐reliant on bank finance and which have a keen interest in retaining access to the UK market. More broadly, future mini‐lateral initiatives could also embrace coalitions where there is alignment with the UK as regards financial governance policy. Indeed, such coalitions could encompass, for instance, the New Hanseatic League, discussed earlier in the article. Depending on the parties’ aspirations, such initiatives could, for instance, implement common principles for future coordination that are supported by sanctions for non‐compliance, or could even go beyond this to introduce legally binding provisions. Moreover, as explored below, all such types of alliance could then have the potential to feed into and ultimately influence the nature and shape of future UK/EU cooperation arrangements. These coalitions could assist in indirectly impacting upon the evolution of future UK/EU financial governance arrangements.^161^

### A functional cooperation arrangement

In this regard, expanding further on the concepts of bilateral and mini‐lateral arrangements, and connecting this to the idea of enhanced differentiation in and around the EU, Pisani‐Ferry et al. have proposed a functional UK/EU Continental Partnership (CP) to sustain economic integration.^162^ While this section suggests that the CP proposal is overly complex and would be unlikely to meet with political approval in the UK, aspects of this model (the concepts of an ‘outer’ and ‘inner’ ring) could be harnessed in order to create a functional UK/EU cooperation arrangement. As the section explores, this could either be a bespoke financial governance framework or part of a broader agreement.

In relation to the original proposal, the authors take as a given that the UK electorate rejected a supranational pooling of sovereignty and the free movement of workers. The proposal advocates a structured outer ring around the EU, which would be of an intergovernmental rather than supranational nature. The outer ring would include the EU, all Member States, the UK plus any other participating states (which could include the EEA states and Switzerland). The authors envisage that the outer ring would be closely involved (although with no final say) in draft EU law making within a CP Council. The outer ring would have to make some sort of contribution to the EU budget and would have to accept the jurisprudence and enforcement mechanisms of the single market (possible via an extended CJEU), including judges from each of the CP states.^163^

The governance surrounding the proposed CP adds layers of complexity to what is an already slow and cumbersome EU legislative process. Moreover, although the CP is focused on economic rather than political integration, given the UK's Brexit policy, the requirements for the UK to pay into the budget, and accept the continued applicability of CJEU jurisprudence are unlikely to gain political traction. Nonetheless, the concepts of an outer and inner ring merit further consideration. As the article's earlier sections identified, heterogeneity characterises and surrounds the EU today. Particularly in a large market (such as financial services), what is needed today is not further top‐down harmonisation and centralised rule‐making but rather a ‘multiplicity of clubs’ providing services that are tailored to the needs and preferences of today's highly diversified polity.^164^ In this regard, there are clear benefits to functional alliances in order to tackle particular transnational problems, which need not then lead to political integration.^165^ For instance, such a framework could be used to develop an improved UK/EU market, which (as is further discussed below), in the first instance could advance a mutually beneficial CMU.

Accordingly, a variant would be for the supranational EU club to form an inner circle, and for there to be a looser compact or outer club surrounding the EU that includes the UK. This outer club would also be capable of developing into a broader, bi‐regional framework with other closely connected satellite states that wished to participate in it (a precedent in this regard is the association agreement between the EU and Mercosur (Argentina, Brazil, Paraguay, and Uruguay)).^166^ In principle this could also evolve into special arrangements and customised institutional structures within the group as regards their various arrangements with the EU.

The outer club would commit, via a bespoke arrangement, to the pursuit of a common policy with the EU on financial governance cooperation (the ‘club goods’). As identified at the outset of this section, while recognising both sides’ autonomy, this arrangement would contain a commitment to pursuing close regulatory (and ideally supervisory) coordination, and for both sides to use best endeavours to maintain mutual access rights as far as possible.^167^ In addition, this broader, bespoke framework could also be influenced and shaped through the emergence and development of bilateral and mini‐lateral alliances explored above, particularly where there are common interests between a number of Member States and the UK as regards financial governance policy. If required, this bespoke UK/EU alliance could also be non‐binding in form.^168^ In line with the article's earlier analysis, soft law mechanisms can still lead to surprisingly effective relationships.^169^ Indeed, this aspect could prove particularly pertinent in the post‐Brexit context, given the current, fractious political landscape where various UK/EU disputes have emerged (including in relation to Northern Ireland, fish, and financial services).^170^ Alternatively however, if sufficient political will develops on both sides over the longer‐term, this arrangement could be implemented via legislation or through putting in place a ‘halfway house’ framework of common principles backed up by remedies in the event of non‐compliance. With respect to concrete projects, given the interdependence of the EU and the UK in the field of financial governance, this alliance could first advance a recalibrated CMU for the benefit of both jurisdictions. Most notably, this could assist in facilitating firms’ straightforward access to market‐based finance in a post Covid‐19 world.^171^

A related aspect to consider is the question of whether the proposed functional cooperation arrangement would be a distinct matter for financial governance, or whether this could form part of a wider agreement. As discussed earlier in the article, the UK/EU TCA contains only very paltry provisions on financial services, and while the TCA does contain provisions on good regulatory practices and regulatory cooperation, these expressly carve out a number of areas including financial regulation. In addition, the accompanying Joint Declaration refers solely to future regulatory cooperation in financial services. Equally, the UK and EU's commitment in the Joint Declaration to agreeing a MoU on such cooperation adopts a light‐touch approach and can be viewed more as a starting point for a future relationship.^172^ However, reflecting back to the 2019 New Political Declaration, this referred to the development of an ambitious, broad and flexible partnership across trade and economic cooperation. The Declaration expressly embraced arrangements on financial services, including with respect to regulatory and supervisory cooperation, as well as envisaging that a future relationship could encompass areas of cooperation beyond those contained in the Declaration.^173^ Further, the 2020 UK Draft Working Text for a Comprehensive UK/EU Free Trade Agreement (FTA) also contained provisions contemplating close and structured regulatory cooperation as regards financial services, as well as including more general provisions on establishing good regulatory practices and regulatory cooperation.^174^ Such language can also be found in other EU FTAs such as in the EU/Japan FTA.^175^ Such provisions indicate that a financial governance cooperation arrangement could be embraced within an umbrella agreement, and that such provisions could also apply more generally.

Accordingly, the short‐term viability of such a functional arrangement, and whether this materialises as a standalone financial services option or is contained within a more generally applicable framework, will be particularly impacted by the evolution of the UK/EU's wider relationship. In this regard, as the article has discussed, the short‐term prospects do not look optimistic. At the time of writing, the immediate political hurdles seem vast, particularly given the current febrile atmosphere and the disagreements which have arisen in fields from fisheries to financial services. Nevertheless the situation could change over the longer term. As the article has articulated, this is not a static situation and the EU's interests can shift over time. Moreover a degree of pragmatism can sometimes be witnessed with the EU, and it will certainly require the UK's markets in relation to its large demands for capital over the next few years (an aspect now accentuated by the pandemic). Given this, the longer‐term prospects for producing a more ambitious framework may be more feasible. In this regard, implementing a more innovative arrangement based on functional cooperation would be an outcome that could prove mutually beneficial to both jurisdictions, and would also be in line with the vision which both sides have previously expressed during earlier phases of the negotiations for a deep and ambitious relationship in this area.^176^

## CONCLUSION

The EU faces a constellation of crises, a situation now exacerbated by the ongoing consequences of the Covid‐19 pandemic. In particular, and as the article has identified, as well as Brexit‐related challenges, there are a number of ongoing internal EU battles, including, but not limited to the financial governance arena. Viewed together, such issues trigger profound questions regarding the future path of the EU project. While it is too early to fully assess whether its current predicaments are a sign that the EU project has gone as far as it can, or whether these are merely diversions on the path to a more integrated Europe,^177^ the current indications certainly point to the EU's resilience to crises, and its aptitude for survival.

Turning to the UK, the early post‐Brexit environment has been beset with economic problems ranging from trade disruptions to the loss of City business to the continent.^178^ Indeed, while the TCA was not intended to tackle financial services in depth, it does little to enable market access for the UK financial sector. In this regard, while the question of future financial services cooperation is being facilitated by the non‐binding MoU (and which is limited to regulatory cooperation),^179^ expectations regarding this are modest at best. This can be understood when one remembers the current political obstacles that are impeding the emergence of a more ambitious new relationship over the shorter term. As the article has discussed, the immediate post‐Brexit UK/EU landscape is one characterised by antagonism between the two jurisdictions on a range of matters, including the row over the supply of the coronavirus vaccine to the continent.^180^ At the same time, Brexit is a process, not a one‐off event, and, with time, this current, febrile atmosphere may dissipate. Moreover the challenges that both the EU and the UK face can be catalysts for creativity and there are precedents, which illustrate the EU's capacity to ‘do things differently’ where this best serves its interests. Indeed, the prospects with respect to a gradual evolution regarding future financial governance relations may be more promising than in other sectors, given that the economic drivers for the UK and EU to cooperate here are particularly strong.

Given this, the article argues that, taking the long‐term view, there is more space for innovation with respect to a future cooperative framework than the current state of affairs would suggest. The article discusses three potential frameworks for new UK/EU regulatory coordination, which could also extend to encompass supervisory cooperation (subject to political consensus). It suggests that joint committees are not a panacea, and that while bilateral and mini‐lateral initiatives are imperfect and limited solutions, such mechanisms remain valuable and could offer some market access to UK firms. It is also probable that such alliances could strengthen the UK's hand with respect to negotiating a new relationship, and that such coalitions could influence the shape of a broader, bespoke UK/EU framework. All such initiatives should therefore be pursued in conjunction with developing a future UK/EU partnership. With respect to this, the article advocates harnessing the dexterous aspects evident within precedents, including existing EU/third country association agreements, in order to develop a functional financial governance arrangement for future cooperation. Such a system would seek to diminish turbulence and would inject new stimulus into projects such as the CMU, an initiative with renewed significance given the Covid‐19 crisis. It could also lead to closer UK/EU cooperation than currently appears likely.

## Footnotes

1 Law and status of post‐Brexit talks stated as of April 2021.

2 ‘Governments Are Spending Big to Keep the World Economy from Getting Dangerously Sick’ *The Economist* 19 March 2020.

3 H. Eidenmüller, 'The Race to Fight Covid‐19: On the Desirability of Regulatory Competition' *Oxford Business Law Blog* 31 March 2020; ‘Nationalism Is a Side Effect of Coronavirus’ *Financial Times* 23 March 2020.

4 J.‐C. Juncker, European Commission Speech, June 2016.

5 See OECD, *Review of International Regulatory Co‐operation of the United Kingdom* (Paris: OECD Publishing, 2020).

6 F. Bruni, ‘Brexit Is Still an Empty Box’ The Future of UK‐EU Financial Services after Brexit, LSE 1 April 2019.

7 See for example ‘Counting the Cost of Brexit's Impact on Trade’ *The Economist* 27 February 2021.

8
*Joint Declaration on Financial Services Regulatory Cooperation between the European Union and the United Kingdom* 24 December 2020 (*Joint Declaration*) 2; HM Treasury, ‘Technical Negotiations Concluded on UK‐EU Memorandum of Understanding’ (26 March 2021).

9 IRSG/Hogan Lovells, *A New Basis for Access to EU/UK Financial Services Post‐Brexit* (September 2017).

10 M. Avbelj, ‘Differentiated Integration – Farewell to the EU‐27?’ (2013) 14 German LJ 191, 211.

11 House of Commons, *UK‐EU Relations after Brexit: An Association Agreement?* Briefing Paper No 8645 (August 2019) 8.

12 See for example High Level Forum on the Capital Markets Union, *A New Vision for Europe's Capital Markets* (June 2020) 4‐6.

13 Financial Centres Futures, *The Global Financial Centres Index 27* (Long Finance & Global Financial Centres, March 2020) 4.

14 House of Commons, *Financial Services: Contribution to the UK Economy* Briefing Paper Number 6193 (July 2019) 5, 9.

15 D. Schoenmaker, *Written Evidence for the House of Lords EU Sub‐Committee on Financial Affairs* (September 2016).

16 House of Lords European Union Committee, *Brexit: Financial Services Report* 9th Report of Session 2016‐17 (December 2016) para 37.

17 O. Wyman, *EU Scenarios and the UK Financial Centre* (2014) 2; European Parliament, *Brexit: The United‐Kingdom and EU Financial Services* (2016) 3‐4.

18 P. Asimakopoulos, 'What Do EU Capital Markets Look Like on the Other Side of Brexit?' *New Financial* September 2019, 3‐4.

19
*ibid*, 4.

20
*ibid*. The report analysed the size and depth of the capital markets in 26 different sectors of activity in all 28 EU countries.

21 W.‐G. Ringe, ‘The Irrelevance of Brexit for the European Financial Market’ Oxford Legal Research Paper, January 2017, 13, referencing Anthony Browne (of the BBA). The Financial Conduct Authority (FCA) data reinforces this, showing approximately 8000 firms registered elsewhere in the EU used the passport to access the UK, while approximately 5,500 UK firms used it to access the EU. The FCA figures also reported that UK firms hold almost 340,000 passports, whereas EU firms hold approximately 23,500, FCA, Letter to Chairman of the Treasury Committee, 17 August 2016, 2‐3. Armour also notes that it is hard to know how to interpret this data given neither measure necessarily shows the activity's economic significance, J. Armour, ‘Brexit and Financial Services’ (2017) 33 *Oxford Review of Economic Policy* S54, 5.

22 M. Kalaitzake, ‘Brexit for Finance? Structural Interdependence as a Source of Financial Political Power within UK‐EU Withdrawal Negotiations’ (2020) *Review of International Political Economy* 1, 11.

23 See for example ‘Brussels Squares up to UK in Fight Over Euro Swaps Clearing’ *Financial Times* 31 March 2021.

24 See ‘The Latest Post‐Brexit Fight Puts Everyone at Risk’ *Bloomberg* 8 March 2021.

25 UK Finance, *Towards a Framework for Financial Services in a UK‐EU Trade Agreement* (November 2017) 3.

26 ‘London's Reign as the World's Capital of Capital Is at Risk’ *The Economist* 29 June 2019; Ernst & Young, *EY Financial Services Brexit Tracker* (June 2019).

27 C. Goodhart and D. Schoenmaker, ‘The Global Investment Banks are now all Becoming American: Does that Matter for Europeans? (2016) 2 *Journal of Financial Regulation* 171.

28 ‘London Bankers Balk at EU Relocation over Virus Travel Worries’ *Financial Times* 28 September 2020.

29 Ernst & Young, *Financial Services Brexit Tracker* (2 March 2021); 'An Update on "Brexit & the City – the Impact So Far"’ *New Financial* October 2019, 3.

30 For instance, in 2016, it was estimated there could be up to 75,000 jobs at risk in a low access scenario, O. Wyman, *The Impact of the UK's Exit from the EU on the UK‐Based Financial Services Sector* (2016) 13; cf an Ernst & Young survey in September 2019 estimated that 1,000 jobs had already been switched and another 7,000 could shift to Europe in the near future, ‘Banks Move Just 1,000 Jobs Despite Brexit Exodus Fears’ *The Times* 20 September 2019.

31 Ernst & Young, n 29 above.

32 See for example FCA, ‘Preparing for Brexit’ 2019 at https://www.fca.org.uk/brexit; FCA, ‘Preparing Your Firm for Brexit’ May 2019 at https://www.fca.org.uk/firms/preparing‐for‐brexit; Bank of England, ‘EU Withdrawal’ 2019 at https://www.bankofengland.co.uk/eu‐withdrawal (all last accessed 26 June 2019).

33 See HM Treasury, *Guidance Document for the UK's Equivalence Framework for Financial Services* (November 2020).

34 The Financial Services (Consequential Amendments) Regulations 2020 (SI 2020/56); FCA, ‘Temporary Permissions Regime’ (2020) at https://fca.org.uk/brexit/temporary‐permissions‐regime‐tpr (last accessed 1 October 2020).

35
*HM Treasury Equivalence Decisions for EEA States* Policy Paper, 9 November 2020.

36 Financial Markets Law Committee, *Addendum to Issues of Legal Uncertainty Arising in the Context of the Withdrawal of the UK from the EU – the Provision and Application of Third Country Regimes in EU Legislation* (November 2019) s 2.1.1. A variety of soft law guidance has been issued, however, including on supporting supervisory convergence in light of Brexit, for example ESMA, *General Principles to Support Supervisory Convergence in the Context of the UK Withdrawing from the EU* ESMA42‐110‐433 (May 2017).

37 See for example ECB, ‘Relocating to the Euro Area’ 2019 at https://www.bankingsupervision.europa.eu/banking/relocating/html/index.en.html (last accessed 26 June 2019).

38 Armour, n 21 above, 5‐6.

39 Limited exceptions to this include, in particular, the temporary reprieve granted to the UK's central counterparties (CCPs) (and can be explained by reference to the potential systemic importance of CCPs in the event a major CCP failed).

40 Commission, 'Slides – Internal EU27 preparatory discussions on the future relationship: "Personal data protection (adequacy decisions); Cooperation and equivalence in financial services"' 10 January 2020; see also Council of the EU, *Annex to Decision Authorising the Opening of Negotiations with the UK for a New Partnership Agreement* 5870/20, February 2020, para 46. Note as well that a 2019 Commission Communication confirms that the EU does not plan to introduce a uniform equivalence assessment and decision‐making process, Commission, *Communication: Equivalence in the Area of Financial Services* COM(2019) 349 Final (July 2019).

41 See for example ‘Amsterdam Punctures City's Post‐Brexit Hopes’ *Financial Times* 11 February 2021.

42 C. Barnard, 'Oral Evidence: Progress of the Negotiations on the UK's Future Relationship with the EU' House of Commons Committee on the Future Relationship with the European Union, June 2020, 11. In addition, if ironing out the more technical details of a new arrangement eventually takes on a lower political salience, this could also impact on the type of partnership that emerges, see for example P.D. Culpepper, *Quiet Politics and Business Power* (Cambridge: Cambridge University Press, 2011); S. James and L. Quaglia, ‘Brexit, the City and the Contingent Power of Finance’ (2019) 24 *New Political Economy* 258, 260.

43 See for example ‘EU Leaders Vent Fears over Delays to Vaccination Drive’ *Financial Times* 25 February 2021.

44 Specifically, as discussed later in the paper, while the UK was a member of the EU, as a non‐euro state, it acted as a key voice for other such non‐euro states. The departure of the UK has altered the balance of power in the Union and has resulted in an enhanced role for euro states, particularly for Germany and France. New groupings are also emerging in response to this. CEPR, *Reconciling Risk Sharing with Market Discipline: A Constructive Approach to Euro Area Reform Policy Insight* No 91 (2018) 3.

45 ‘Nine Eurozone Countries Issue Call for “CoronaBonds”’ *Financial Times* 25 March 2020.

46 ‘EU Recovery Fund: How the Plan Will Work’ *Financial Times* 21 July 2020. Indeed the protracted debates in the run‐up to the compromise exposed the continuing fissures, and suggests that the political conditions for full fiscal coordination in the eurozone do not yet exist.

47 ECON, *An Assessment of the Impact of Brexit on Euro‐Area Stability* IP/A/ECON/NT/2016‐04 (28 November 2016) 13, 25.

48 T. Sampson, *Brexit: The Economics of International Disintegration* Vox CEPR Policy Portal (2017).

49 Commission, *White Paper on the Future of Europe* COM(2017) 2025 Final (2017).

50 See S. Hix and N. Sitter, *Svexit or Huxit? How Another Country Could Follow the UK out of the EU* LSE Brexit Blog, January 2018; T. Oliver, *European and International Views of Brexit* (2016) 4.

51 ‘UK's Small Businesses Struggle with Brexit Red Tape’ *Financial Times* 28 January 2021; Hix and Sitter, *ibid*.

52 See G. Majone, ‘The European Union Post‐Brexit: Static or Dynamic Adaptation?’ (2017) 23 *European Law Journal* 9, 25‐27.

53 F. Jacobs, *The EU after Brexit* (Palgrave Macmillan, 2018) ch 2; Commission, n 49 above.

54 Eidenmüller, n 3 above.

55 ‘Nationalism Is a Side Effect of Coronavirus’ n 3 above; see also for example D. Rodrik, ‘What's Driving Populism?’ *Social Europe* 23 July 2019.

56 See for example ‘Nine Eurozone Countries Issue Call for Coronabonds’ *Financial Times* 21 July 2020.

57 ‘EU Circles Wagons Against Criticism of Covid Vaccine Rollout’ *Financial Times* 5 February 2021.

58 ‘Eurozone Countries Strike Emergency Deal on Coronavirus Rescue’ *Financial Times* 9 April 2020; ‘EU Recovery Fund: How the Plan Will Work’ *Financial Times* 21 July 2020.

59 N. Moloney, ‘Panel Discussion’ The Future of UK‐EU Financial Services after Brexit, LSE 1 April 2019.

60 Commission, *Communication on Further Steps Towards Completing Europe's EMU: A Roadmap* COM(2017) 821 Final (2017) 11; Commission, *A Blueprint for a Deep and Genuine Economic and Monetary Union* COM(2012) 777 Final (2012) 30.

61 B. Eichengreen, ‘The International Financial Implications of Brexit’ (2019) 16 *International Economics and Economic Policy* 37, 45.

62 IMF, *The Quest for Lasting Stability: GFSR Report* (Washington, DC: IMF, 2012) 56; M. Chang, ‘Brexit and the EU Economic and Monetary Union’ in F. Fabbrini (ed), *The Law & Politics of Brexit* (Oxford: OUP, 2017) 166. Specifically, many banks in the euro area hold large amounts of domestic government debt, and when states provide public assistance to struggling banks, states increase their own indebtedness, which devalues the government bonds held by the banks, see D. Schäfer, ‘A Banking Union of Ideas? The Impact of Ordoliberalism and the Vicious Circle on the EU Banking Union’ (2016) 54 *Journal of Common Market Studies* 961, 970.

63 Commission, *Banking Union: Restoring Financial Stability in the Eurozone: Updated Memo* (9 March 2015).

64 Note that the European Stability Mechanism can also recapitalise eurozone banks directly if particular conditions are satisfied.

65 D. Howarth and L. Quaglia, ‘The Difficult Construction of a European Deposit Insurance Scheme: A Step Too Far in Banking Union?’ (2018) 21 *Journal of Economic Policy Reform* 190, 194.

66 A. Mullineux, ‘"Brexit": "The City" and EU Capital Markets’ (16) 1 I*nternational Economics and Economic Policy* 17, 24. There is also the concern regarding creating moral hazard for those that could draw on EU funds to support their own national schemes, see Howarth and Quaglia, *ibid*, 194. Moreover, recent proposals introduced with a view to breaking deadlock have met with opposition from Italy and other states due to the conditions attached and countries remain sharply divided, see for example O. Scholz, ‘Germany Will Consider EU‐Wide Bank Deposit Reinsurance’ *Financial Times* 5 November 2019.

67 Bulgaria, Denmark, Croatia, Poland, Romania, Sweden, the Czech Republic, and Hungary. Note that in July 2020, Bulgaria and Croatia established close cooperation arrangements between the ECB and their central banks, and they acceded to the Single Supervisory and Single Resolution Mechanisms from October 2020.

68 In particular the UK engineered a revised voting system in the European Banking Authority; a double simple voting majority system of Member States inside and outside the euro area to prevent non‐euro states being overwhelmed by euro banking decisions, M. Chang, *Can Brexit Lead to Further Integration? The Case of Economic and Monetary Union* College of Europe Policy Brief 6.17 (June 2017) 1.

69 P. Tokarski and S. Funk, *Non‐Euro Countries in the EU after Brexit* SWP Comment No 3 January 2019, 3. The double voting rule is also set to fall away once four of the EU‐8 join the Single Supervisory Mechanism.

70 Chang, n 68 above, 2; Oliver, n 50 above, 3 (who notes that, for instance, newer members such as Bulgaria have concerns that Brexit could marginalise them further outside the eurozone).

71 M. Brunnermeier, H. James and J.‐P. Landau, *The Euro and the Battle of Ideas* (Princeton, NJ: Princeton University Press, 2016), 2‐3 (drawing on Max Weber's work who thought conceptual clarification could assist in better comprehending problems, debates and institutions).

72 Tokarski and Funk, n 69 above, 4.

73 The eight are Denmark, Estonia, Finland, Ireland, Latvia, Lithuania, the Netherlands, and Sweden.

74 Hanseatic League, 'Finance Ministers Underline Their Shared Views and Values in the Discussion on the Architecture of the EMU ' March 2018.

75 Commission, *Action Plan on Building a Capital Markets Union* COM(2015) 468 Final (2015).

76 D. Howarth and L. Quaglia, ‘Brexit and the Single European Financial Market’ (2017) 55 *Journal of Common Market Studies* 149, 152; U. Koltz and J. Schild, ‘Back to the Future? Franco‐German Bilateralism in Europe's Post‐Brexit Union’ (2018) 25 J*ournal of European Public Policy* 1174, 1180.

77 W. Wright, 'What Next for European Capital Markets?' *New Financial* June 2016, 2; P. Ständer, *What Will Happen to the CMU after Brexit* Policy Paper 181, Jacques Delors Institut (December 2016) 3.

78 See for example ‘Brussels Squares up to UK in Fight Over Euro Swaps Clearing’ *Financial Times* 31 March 2021.

79 Commission, *Capital Markets Union – Accelerating Reform* COM(2016) 601 Final (2016); Commission, *Communication on CMU: Progress on Building a Single Market for Capital for a Strong EMU* COM(2019) 136 Final (2019); see also Commission, *Communication: A Capital Markets Union for People and Businesses – New Action Plan* COM(2020) 590 final (2020).

80 W.‐G. Ringe, ‘The Politics of Capital Markets Union: From Brexit to Eurozone’ in F. Allen and others (eds), *Capital Markets Union and Beyond* (Cambridge, MA: MIT Press, 2019) 9; House of Lords European Union Committee, n 16 above, paras 29‐37.

81 N. Moloney, ‘Capital Markets Union, Third Countries, and Equivalence’ in D. Busch, E. Avgouleas and G. Ferrarini (eds), *Capital Markets Union in Europe* (Oxford: OUP, 2018) 128; Moloney, n 59 above.

82 Moloney, ‘Capital Markets Union, Third Countries, and Equivalence’ *ibid*, 130; E. Ferran, ‘The UK as a Third Country Actor in EU Financial Services Regulation’ (2017) 3 J*ournal of Financial Regulation and Compliance* 40.

83 Moreover, as discussed earlier in the article, in 2019 the EU refused to extend a decision on Switzerland's stock market equivalence; and it withdrew equivalence status from five other countries regarding credit rating regulation.

84 Majone, n 52 above, 12.

85 W.‐G. Ringe, ‘Capital Markets Union for Europe: A Commitment to the Single Market of 28’ (2015) 9 *Law and Financial Markets Review* 5, 17‐18.

86 S. Weatherill, ‘The Several Internal Markets’ (2017) 36 *Yearbook of European Law* 125.

87 A. Menon, ‘An Inflexible Brussels Is Damaging Its Own Interests over Brexit’ *Financial Times* 19 November 2019.

88 House of Lords European Union Committee, *Brexit: The Options for Trade* 5th Report of Session 2016‐17 (December 2016) ch 5; M. Emerson, *Which Model for Brexit?* CEPS Special Report No 14 (October 2016) 6, 14.

89 HM Government, *Which Model for Brexit? Possible Models for the UK Outside the EU* (March 2016) 26.

90 Ringe, n 21 above, 23.

91 E. Ferran, ‘European Banking Union: Imperfect, but It Can Work’ Cambridge Faculty of Law Research Paper No 30/2014 at https://papers.ssrn.com/sol3/papers.cfm?abstract_id=2426247 (last accessed 20 July 2018).

92 Ringe, n 21 above 26.

93 C. Hobley, 'Reflections on Brexit ‐ the Road to Here and the Paths Ahead' University of Cambridge, Centre for European Legal Studies Lunchtime Seminar, 20 November 2019.

94 There is also the enhanced cooperation procedure enabling a caucus of Member States to pursue positive harmonisation projects, such as the proposed Financial Transaction Tax.

95 Avbelj, n 10 above, 210‐211.

96 New Political Declaration Setting out the Framework for the Future Relationship between the European Union and the United Kingdom (19 October 2019) para 37. As discussed below, cooperation would include, on the one side, the UK regulators (the Prudential Regulation Authority and the Financial Conduct Authority) and on the other, the EU supervisory authorities.

97 HM Treasury, *Call for Evidence: Regulatory Coordination* (July 2019) para 1.2.

98 Specifically, expanding cross‐border investment channels can enhance the markets’ competitiveness; and agreeing arrangements for cross‐boundary regulatory and enforcement cooperation signifies their commitment to strengthen enforcement cooperation; see for example Dorsey & Whitney, *Guide to China‐Hong Kong Stock Connect* (2016).

99 Trade and Cooperation Agreement between the European Union and the European Atomic Energy Community, of the One Part, and the United Kingdom of Great Britain and Northern Ireland, of the Other Part (24 December 2020).

100 See TCA, *ibid*, s 5 for the specific provisions included on cross‐border financial services.

101
*Joint Declaration* n 2 above, 2; HM Treasury, n 2 above.

102 Barnard, n 42 above, 11.

103 A. Duff, 'Brexit: Terra Nova to Explore Together' EPC Discussion Paper 7 November 2017, 5.

104 House of Commons, n 11 above, 8, 30.

105 Bruni, n 6 above.

106
*The New Political Declaration on the Future Relationship*, paras 35 and 37.

107 See CMS Legatum Institute, *A New UK/EU Relationship in Financial Services* (April 2017) ch 6.

108 ESFRC, *How to Mitigate the Shock of Brexit to Financial Services in the EU* Statement No 46 (April 2019).

109 IRSG/Hogan Lovells, n 9 above, 9.

110 ESFRC, n 108 above.

111 Agreement on the Withdrawal of the United Kingdom of Great Britain and Northern Ireland from the European Union and the European Atomic Energy Community (19 October 2019), Arts 164‐166. *The New Political Declaration on the Future Relationship* also proposes establishing a joint committee for managing and supervising the operation of the future relationship, paras 126‐127.

112 Agreement on the European Economic Area (OJ No L 1, 3.1.1994, 3 and EFTA States’ official gazettes), Arts 92‐94.

113 Comprehensive and Economic Trade Agreement (provisionally in force September 2017), Art 26.1; Art 26.2.1(f)). See also the Agreement between the EU and Japan for an Economic Partnership (1 February 2019).

114 EFTA‐Ukraine Free Trade Agreement (June 2012), Art 8.

115 EU representation includes the Commission, the ECB, and (with respect to some ISSBs), ESMA; N. Moloney, ‘International Financial Governance, the EU, and Brexit: The ‘Agencification’ of EU Financial Governance and the Implications’ (2016) 17 EBOR 451, 452.

116
*ibid*, 453, 473‐474. Equally though, the ESAs will lose the technical expertise from UK regulators who are well‐versed in working with a complex and evolving financial system.

117 CETA, Arts 26 and 29; TCA, Part Six, Title I. See in this regard also the UK Draft Working Text for a UK‐EU Comprehensive Free Trade Agreement (19 May 2020), Art 17.17 (financial services dispute settlement) and ch 33 (dispute settlement).

118 LSE Conference: Implications of Brexit for the Governance of UK‐EU Financial Services, 1 April 2019.

119 See Regulation (EU) No 600/2014 on Markets in Financial Instruments and amending Regulation (EU) No 648/2012 (MiFIR), Art 46(4) (as amended by Regulation (EU) 2019/2033 on the Prudential Requirements of Investment Firms, Art 63(4)(b)),

120 Accordingly, Member States will apply at least the minimum standards that apply to branches established in other Member States, see Directive 2013/36/EU on Access to Activity of Credit Institutions and Investment Firms, Art 47.

121 Directive 2011/61/EU on Alternative Investment Fund Managers and amending Directives 2003/41/EC and 2009/65/EC and Regulations (EC) No 1060/2009 and (EU) No 1095/2010, Art 42.

122 FINMA Communication, 6 January 2014 at https://www.finma.ch/en/authorisation/institutions‐and‐products‐subject‐to‐the‐collective‐investment‐schemes‐act/notification‐procedure/ (last accessed 2 March 2021).

123 IOSCO, *Market Fragmentation and Cross‐Border Regulation* (June 2019) 24.

124 C. Brummer, ‘Post‐American Securities Regulation’ (2010) 98 *California Law Review* 327, 367.

125 SEC, *Cooperative Arrangements with Foreign Regulators* (March 2017) (including supervisory and enforcement cooperation arrangements as well as technical assistance).

126 E. Cadmus, ‘Revisiting the SEC's Memoranda of Understanding: A Fresh Look’ (2011) 33 *Fordham International Law Journal* 1800, 1839, 1853.

127
*Amended and Restated Memorandum of Understanding Concerning Consultation, Cooperation and the Exchange of Information Related to Market Oversight and the Supervision of Financial Services Firms* (29 March 2019); US Securities and Exchange Commission and the UK Financial Services Authority, *Memorandum of Understanding Concerning Consultation, Cooperation and the Exchange of Information related to Market Oversight and the Supervision of Financial Services Firms* (14 March 2006).

128
*Amended and Restated Memorandum of Understanding ibid* recitals, Art 2, para 11; FCA, *The UK Financial Conduct Authority and the US Securities and Exchange Commission sign Updated Supervisory Cooperation Arrangements* (29 March 2019).

129
*Amended and Restated Memorandum of Understanding ibid*, see for example recitals, Art 2, para 2, and Art 5.

130 J. Hill, ‘Regulatory Cooperation in Securities Market Regulation: Perspectives from Australia’ (2020) 17 *European Company and Financial Law Review* 11.

131 Cadmus, n 126 above, 1855; L. Bromberg, A. Godwin and I. Ramsay, ‘Cross‐Border Cooperation in Financial Regulation: Crossing the Fintech Bridge’ (2018) 13 *Capital Markets Law Journal* 59, 63‐64.

132 Brummer, n 124 above, 337‐338.

133 Cadmus, n 126 above, 1828.

134 See for example ‘After Brexit: the UK and EU Risk a State of “Permanent Alert”’ *Financial Times* 18 February 2021.

135 M. Lehmann and D. Zetzsche, ‘Brexit and the Consequences for Commercial and Financial Relations between the EU and the UK’ (2016) 27 *European Business Law Review* 999, 1018‐1019.

136
*ibid*, 1019.

137 CMS Legatum Institute, n 107 above, 85‐86.

138 FCA, *FCA Agrees MoUs with ESMA and EU Regulators to Allow Cooperation and Exchange of Information* (February 2019); FCA, *FCA Confirms MoUs with ESMA and EU Securities Regulators* (17 July 2020).

139 Financial Supervisory Authority (FIN‐FSA), *Brexit and Third Country Firm Cross‐Border Authorisation Regarding Investment Services* (15 September 2020); Allen & Overy, *EEA Transitional Measures Tracker* (September 2020).

140 As discussed above, MiFIR, Art 46(4) provides that Member States may allow third‐country firms to provide investment services in the absence of a Commission equivalence decision, see also Clifford Chance, *The Netherlands/Brexit: Exemption Regulation Extended* (February 2019).

141 ‘Britain and the EU Will Remain Close Even after Brexit’ *The Economist* 31 October 2019.

142 FCA, *The FCA and the Dutch Authority for the Financial Markets (AFM) Agree on Closer Partnership* (June 2019).

143 D. Henig, *Sweden, UK and the EU: Managing Post‐Brexit Relations and Defining a New Agenda for European Competitiveness* ECIPE Policy Brief No 1/2019 (February 2019) 23.

144
*ibid*, 12.

145 General Affairs Council Meeting, ‘Council Conclusions on a Homogenous Extended Single Market and EU Relations with Non‐EU Western European Countries’ (16 December 2014).

146 Commission, *Communication: Equivalence in the Area of Financial Services* n 39 above, 1.

147 Commission, *Building a Capital Markets Union: Green Paper* COM(2015) 63 final, 8.

148 Goodhart and Schoenmaker, n 27 above, 172‐173.

149 C. Brummer, *Minilateralism* (Cambridge: CUP, 2014) 1, 144‐145.

150
*ibid*, ch 1; E. Moret, 'Effective Minilateralism for the EU: What, When and How' EUISS June 2016.

151 Brummer, n 149 above, ch 1; Moret, *ibid*.

152 German Development Institute, *Between Minilateralism and Multilateralism: Opportunities and Risks of Pioneer Alliances in International Trade and Climate Politics* Briefing Paper 16/2015.

153 Moret, n 50 above, 1; see for example C. Creamer, ‘From the WTO's Crown Jewel to Its Crown of Thorns’ (2019) 113 *American Journal of International Law* 51.

154 Moret, *ibid*.

155 Brummer, n 149 above, 1‐2; Moret, *ibid*, 2.

156 Moret, *ibid*, 3.

157 D. Howarth and L. Quaglia, ‘Brexit and the Battle for Financial Services’ (2018) 25 J*ournal of European Public Policy*, 1118, 1131; S. Lavery, S. McDaniel and D. Schmid, ‘Finance Fragmented? Frankfurt and Paris as European Financial Centres after Brexit’ (2018) 25 J*ournal of European Public Policy* 1, 3; H. Farrell and A. Newman, ‘The New Interdependence Approach: Theoretical Development and Empirical Demonstration’ (2016) 23 *Review of International Political Economy* 713, 716, 723.

158 Howarth and Quaglia, *ibid*, 1121 (noting that this explanation fits well with explanations from the Bank of England and the UK‐based finance industry); Lavery, McDaniel and Schmid, *ibid*, 3.

159 Howarth and Quaglia, *ibid*, 1119‐1120, 1130.

160 James and Quaglia, n 42 above, 267‐268.

161 L. Quaglia, ‘Brexit and EU Financial Regulation’ in P. Diamond, P. Nedergaard and B. Rosamond (eds), *The Routledge Handbook of the Politics of Brexit* (Abingdon: Routledge, 2018) 249.

162 J. Pisani‐Ferry and others, *Europe after Brexit: A Proposal for a Continental Partnership* (Bruegel, August 2016) 6.

163
*ibid*, 7.

164 Majone, n 52 above, 22‐23.

165
*ibid*, 23. Examples such as US‐Canada relations, and the Benelux, demonstrate that economic and political integration are different processes.

166
*Interregional Framework Cooperation Agreement between the EC, Its Member States and the Southern Common Market and Its Party States* (1995).

167 CMS Legatum Institute, n 107 above, 74.

168 See for example M. Barnier, *Speech at the Eurofi General Assembly* 30 June 2020 (suggesting that future cooperation should be a voluntary framework for dialogue among regulators and supervisors).

169 In the event an agreement can be concluded, a dispute settlement system would be required to ensure the arrangements continue to conform to agreed principles. Rather than revisiting the various red lines regarding the CJEU, inspiration could, for instance, be drawn from the EFTA docking model, which could be tweaked to the UK/EU scenario.

170 See for example ‘After Brexit: the UK and EU Risk a State of “Permanent Alert”’ *Financial Times* 18 February 2021.

171 High Level Forum on the Capital Markets Union, n 12 above.

172 See TCA, s 5; *Joint Declaration*, n 2 above, 2.

173
*The New Political Declaration on the Future Relationship*, paras 3; 17; 28, 35‐37.

174 UK Draft Working Text for a UK‐EU Comprehensive Free Trade Agreement, n 117 above, Art 17.19; ch 25.

175 Agreement between the EU and Japan for an Economic Partnership, n 113 above, Art 8.67 which states, ‘[t]he Parties shall promote regulatory cooperation on financial regulation …’ and Annex 8‐A which provides the nuts and bolts of such regulatory cooperation (including its scope, the applicable principles and framework for regulatory cooperation, the details of the joint EU/Japan financial regulatory forum, and provisions on dispute resolution); see also Chapter 18 on ‘Good Regulatory Practices and Regulatory Cooperation’ (specifically, the chapter's objectives have the general aim of enhancing bilateral trade and investment); D. Frost, Letter to Michel Barnier, 19 May 2020.

176 For instance the Commission President has formerly advocated a partnership of high ambition that goes beyond trade, is unprecedented in scope, and includes financial services; and the UK Government has also expressly referred to the ‘depth of the relationship in this area’ and that there should be enhanced provision for regulatory and supervision cooperation arrangements; see for example U. von der Leyen, 'Old Friends, New Beginnings: Building Another Future for the EU‐UK Partnership' Speech at the LSE, London, 8 January 2020, Commission, n 40 above, 20‐21; B. Johnson, ‘UK/EU Relations’ Written Statement, HCWS86 3 February 2020. See also HM Government, *The Future Relationship with the EU: The UK's Approach to Negotiations* CP211 (February 2020), paras 53‐55; see also Bruni, n 6 above.

177 Sampson, n 48 above, 181‐182.

178 See for example ‘Amsterdam Ousts London as Europe's Top Share Trading Hub’ *Financial Times* 10 February 2021.

179 HM Treasury, n 2 above.

180 ‘Ursula von der Leyen Feels the Heat over Vaccine Exports Hiccup’ *Financial Times* 31 January 2021.

